# 

**Published:** 1894-03

**Authors:** 


### TABLE OF CONTENTS.

ORIGINAL COMMUNICATIONS. PAGE

The Scallop Wire Plate for retaining Teeth in Place after widening the Dental Arch, and for correcting Slight Irregularities when Necessary. By J. N. FARRAR, M.D., D.D.S., New York City . . . . . . . . . . . . 145

The Care of the Teeth to the Fifteenth Year. By SAFFORD G. PERRY, D.D.S., New York City . . . . . . . . . . . . . . . . . . . . . . . 148

Foods. By PROFESSOR R. W. GREENLEAF . . . . . . . . . . . . . . . 163

Physiological and Pathological Changes in the Alveolar Tissues, from Operations for Correction of Irregularities of Teeth. By DR. J. N. FARRAR . 172

REPORTS OF SOCIETY MEETINGS.

Odontological Society of Pennsylvania . . . . . . . . . . . . . . . . . . . 173

Harvard Odontological Society . . . . . . . . . . . . . . . . . . . . . . . 187

EDITORIAL.

Another Advance is Necessary . . . . . . . . . . . . . . . . . . . . . . . 194

No more need apply . . . . . . . . . . . . . . . . . . . . . . . . . . . . 197

BIBLIOGRAPHY . . . . . . . . . . . . . . . . . . . . . . . . . . . . . . 198

OBITUARY.

Dr. Samuel J. Dickey . . . . . . . . . . . . . . . . . . . . . . . . . . . 198

DOMESTIC CORRESPONDENCE.

Pulp Nodules . . . . . . . . . . . . . . . . . . . . . . . . . . . . . . . 199

NOTES AND COMMENTS . . . . . . . . . . . . . . . . . . . . . . . . . 200

CURRENT NEWS.

The General Medical Council of Great Britain . . . . . . . . . . . . . . 203

New Jersey State Dental Society . . . . . . . . . . . . . . . . . . . . . 204

Ohio State Dental Society . . . . . . . . . . . . . . . . . . . . . . . . . 205

Woman's Dental Association of the United States . . . . . . . . . . . . 205

St. Louis Dental Society . . . . . . . . . . . . . . . . . . . . . . . . . 205

International Medical Congress . . . . . . . . . . . . . . . . . . . . . . 206

Illinois State Dental Society . . . . . . . . . . . . . . . . . . . . . . . 206

Recent Patents . . . . . . . . . . . . . . . . . . . . . . . . . . . . . . . 206

SELECTIONS.

Creosotal . . . . . . . . . . . . . . . . . . . . . . . . . . . . . . . . . 207

The Antidote for Arsenic . . . . . . . . . . . . . . . . . . . . . . . . . 207

Thiocamf . . . . . . . . . . . . . . . . . . . . . . . . . . . . . . . . . 208

